# eHealthResp, a Digital Intervention to Improve Antibiotic Prescribing in Respiratory Infections: A Pilot Study

**DOI:** 10.3390/life12081160

**Published:** 2022-07-30

**Authors:** Tânia Magalhães Silva, Marta Estrela, Sandra Magalhães, Catarina Simões, Afonso Cachim, Tainá Costa, Gabriella Crexinski, Margarida Pisco Almeida, Adolfo Figueiras, Fátima Roque, Maria Teresa Herdeiro

**Affiliations:** 1 iBiMED—Institute of Biomedicine, Department of Medical Sciences, University of Aveiro, 3810-193 Aveiro, Portugal; mestrela@ua.pt (M.E.); sandra.vicencia@ua.pt (S.M.); 2 Department of Medical Sciences, University of Aveiro, 3810-193 Aveiro, Portugal; catarina.simoes@ua.pt (C.S.); tainacosta@ua.pt (T.C.); gabriellacrexinski@icloud.com (G.C.); 3 Faculty of Medicine, University of Porto, 4200-319 Porto, Portugal; afonsocachim98@gmail.com; 4 Department of Communication and Art/DigiMedia, University of Aveiro, 3810-193 Aveiro, Portugal; marga@ua.pt; 5 Department of Preventive Medicine and Public Health, University of Santiago de Compostela, 15782 Santiago de Compostela, Spain; adolfo.figueiras@usc.es; 6 Health Research Institute of Santiago de Compostela (IDIS), University of Santiago de Compostela, 15706 Santiago de Compostela, Spain; 7 Consortium for Biomedical Research in Epidemiology and Public Health (CIBER Epidemiology and Public Health—CIBERESP), 15706 Santiago de Compostela, Spain; 8 Research Unit for Inland Development, Guarda Polytechnic Institute (UDI-IPG), 6300-559 Guarda, Portugal; 9 Health Sciences Research Center, University of Beira Interior (CICS-UBI), 6201-001 Covilhã, Portugal

**Keywords:** antibiotic resistance, e-health, online course, mobile application, respiratory tract infections, physicians

## Abstract

The emergence of antibiotic resistance (ABR) is one of the most serious public health threats worldwide. The inappropriate use of antibiotics is considered the main determinant for the increase and development of ABR, contributing to a greater risk of therapeutic ineffectiveness, particularly within primary care context. Therefore, this pilot study aims to raise awareness and promote an adequate antibiotic use among physicians, through the evaluation of the eHealthResp platform, a digital intervention composed by an online course and a mobile application, to aid in the management of respiratory tract infections. The global validation of the eHealthResp platform was carried out by 12 physicians who explored and performed a quantitative and qualitative evaluation of the contents of the online course and mobile app. The global evaluation of the analyzed parameters was very positive, with the highest median scores being attributed to adequacy, correction, format, and trust of the eHealthResp platform. The qualitative feedback enhanced the contents’ relevance, clarity, and consolidation, as well as the effectiveness of the educational intervention against ABR. Overall, this study revealed that the eHealthResp may be regarded as an important e-health tool for the management of respiratory tract infections and improvement of antibiotic prescription practices among physicians.

## 1. Introduction

Since their discovery, more than 90 years ago, antibiotics have been seen as a group of essential drugs in the treatment and cure of several bacterial infections [1]. Over time their use has grown exponentially, and if this pattern continues, antibiotic resistance (ABR) will become a bigger issue than it already presents itself as [2]. The inadequate use of antibiotics has been exponentially growing over time, leading to ABR, which is currently one of the 10 major threats to public health worldwide [3]. A 2019 study performed in 204 countries estimated that ABR caused 1.27 million deaths, a higher death count than in other diseases, like malaria or HIV/AIDS [4]. This public health challenge has been tackled under the World Health Organization (WHO) One Health approach, as this threat involves the interaction between humans, animals, and the surrounding environment [5].

Upper and lower respiratory tract infections are responsible for increased mortality rates, being the world’s third leading cause of death, with 4.25 million deaths worldwide in 2010 [6], causing 17–22% of all deaths in children with less than 5 years and 3% of deaths in adults aged from 15 to 49 years [7]. Antibiotics are known to be commonly used to treat respiratory infections, with nearly three quarters of antibiotic prescriptions targeting upper respiratory tract cases [8]. However, as most of these infections often have a viral origin instead of a bacterial one, an increase in antibiotic prescription rates, twice as much as expected, has been observed [9,10].

The unnecessary prescriptions in the long run will cause an increase in ABR since the prescribing errors mostly stem from a misdiagnosis of the origin of the infection, which may increase patients’ length of stay in the hospital, therefore, promoting unwise allocation of necessary hospital resources [9,11].

The major goals of many public health policies regarding the subject of antimicrobial resistance in respiratory tract infections are to save as many new antibiotics from use as possible, so that eventually these can be used without the risk of microbial agents developing resistance [1,12]. However, these goals cannot be achieved without prior work, requiring both incentives for research and the creation of interventions in public health, in order to change policies and legislation [13].

Various interventions aiming to minimize the prescription of antibiotics have already been put in place, with educational materials targeting primary care physicians having achieved the best results in reducing the use of antimicrobial drugs [14,15,16,17,18,19]. Clinical guidelines are also being developed and implemented in order to assist physicians in the decision of what drug to use, taking into account the WHO Access, Watch, Reserve (AWaRe) classification put in place by the WHO and choosing a narrow-spectrum antibiotic [20]. Only by being fully informed and aware of the danger that ABR poses for public health can health professionals make sound decisions with their patient’s best intent in mind. It is of extreme importance that healthcare professionals have at their disposal the best and most reliable information, so they can make informed decisions about the treatment method for their patients, by prescribing the most adequate therapeutic options available without putting public health in general at risk [21].

With all this in mind, we developed the eHealthResp project, which includes a digital platform composed of two online courses, especially targeted for primary care physicians and community pharmacists, and a mobile application. These digital platforms include several presentations with state-of-the-art information about respiratory infections and algorithms addressing respiratory infections management, with the ultimate goal of promoting the appropriate use of antibiotics to treat infections of the respiratory tract.

The main goal of this study is to evaluate the feedback obtained from physicians after exploring the digital platforms through a pilot study. This evaluation will help to identify the platforms’ strengths and weaknesses while providing feedback about the functionality of eHealthResp, which will improve both the platform’s accessibility and appropriateness to the health professionals who use it as a clinical decision support tool for the treatment of respiratory infections.

## 2. Methods

### 2.1. Setting

The eHealthResp project, composing of an educational intervention designed for health professionals, including primary care physicians and community pharmacists, primarily aims to evaluate the effectiveness of e-Health tools to support health professionals’ decisions in the management of respiratory tract infections, thus improving patients’ health outcomes. The educational intervention comprises the: (i) eHealthResp online course, developed with a user-centred design and composed of 6 or 4 modules (respectively for physicians and pharmacists), and 4 clinical cases; and (ii) eHealthResp clinical decision support system, in the form of a mobile application available for Android and iOS systems, which will be carried out through a cluster randomized controlled trial on the catchment area of Portugal’s Centre Regional Health Administration (ARS-C).

Both the courses and the mobile application have been previously validated by experts in relation to their contents (including the clinical cases) by using the Delphi Method approach [22], as well as for their usability, through usability testing [23,24], thus highlighting its user-friendliness, consistency, and usefulness.

However, before the educational intervention takes place, a pilot study was conducted in a small group of health professionals from the geographical area of Portugal’s North Regional Health Administration (ARS-N).

### 2.2. Pilot Study

Twelve physicians working in the catchment area covered by the ARS-N were invited to participate in this pilot study via e-mail and to explore the contents of both the online course and the mobile application. A convenience sample was used and, before invitation to participate in the study, signed consents were obtained from all participants, prior to use of their e-mail contacts, where each participant was informed about the objectives and scope of this study and freely consented to participate. In agreement with the General Data Protection Regulation (GDPR), before assessing the online course and exploring the mobile application, each physician had previously given their informed consent allowing the sending of the link, by e-mail, with the access to the online course modules, clinical cases, and to a final evaluation questionnaire, as well as a link to download the mobile application and create the password for using the app. Participants had complete autonomy to explore and evaluate the eHealthResp online course and mobile application, and their anonymity was completely safeguarded.

#### 2.2.1. eHealthResp Online Course

The eHealthResp online course (see Supplementary Material S1), targeted for primary care physicians and developed with a user-centered design, is composed of 6 modules providing clinical information on several respiratory tract infections, such as, acute otitis media, acute rhinosinusitis, acute pharyngotonsillitis, acute tracheobronchitis, community-acquired pneumonia, and COVID-19, together with a final evaluation composed of 4 clinical cases to be solved after completing the modules [22,24].

#### 2.2.2. eHealthResp Mobile Application

The eHealthResp mobile app is an e-health tool developed to assist primary care physicians in advising patients in cases of respiratory tract disorders (see Supplementary Material S2). It includes a physician profile composed of five algorithms based on the suspected disease: (i) acute otitis media, (ii) acute rhinosinusitis, (iii) acute pharyngotonsillitis, (iv) acute tracheobronchitis, and (v) community-acquired pneumonia, and guides the most likely diagnosis and potential therapeutic approaches, based on respiratory symptoms [23,25].

### 2.3. Global Content Validation Questionnaire

The global validation of the eHealthResp online course and mobile application consisted in the invitation of the participants to complete a final questionnaire, after undertaking the online course and exploring the app, which comprises three main sections of questions: (1) sociodemographic data (composed of five brief questions addressing gender, age, education level, medical specialty, and years of experience); (2) four groups of closed questions, aiming to quantitatively evaluate the contents and elements of the online course [22], clinical cases, and mobile application; and (3) four open-answer questions, aiming to qualitatively evaluate the online course, the mobile application, and the educational intervention, by analyzing the suggestions and comments provided by the participants.

### 2.4. Statistical Analysis

Descriptive statistical analyses were employed to illustrate demographic characteristics and to quantitatively evaluate the contents of the eHealthResp online course and mobile application. Results were reported as mean ± standard deviation (SD), median, and 25th and 75th percentiles. Additionally, in order to gain a better insight about the participant’s opinions, comments, and suggestions overall, as well as to clarify possible comprehensible problems and comment on any difficulties experienced, the research team also evaluated the qualitative feedback provided by the physicians.

### 2.5. Ethics Statement

This pilot study was approved by the Guarda Polytechnic Institute’s Ethics Committee (code number: 7/2021). The compliance with the provisions of the General Data Protection Regulation-Directive 95/46/EC (GDPR) was ensured, guaranteeing the security, anonymity and confidentiality of all data provided by the participants. Participation in the study was voluntary and participants provided their informed consent before participation.

## 3. Results

Twelve physicians consented to participate on this pilot study, to explore both digital platforms, and to answer the questionnaire.

### 3.1. Sociodemographic Characteristics of the Physicians

Based on the data retrieved from the first section of the questionnaire, a few sociodemographic characteristics were collected from each physician. Of the 12 participants included in this study, 5 were female and 7 were male. The mean age of all participants was 38.75 (±13.42) years old. The majority of the physicians had a master’s degree (*n* = 8) and only three had a PhD. In relation to the medical specialty, more than half of the participants were primary care physicians (*n* = 7). The average years of medical experience was 10.83 (±13.65).

### 3.2. Evaluation of the eHealthResp Online Course

The global evaluation performed by the physicians of the online course parameters, namely those obtained from the modules and clinical cases, is displayed in Table 1.

All 12 participants who answered the questionnaire evaluated the online course, in general, with a mean score of 4.46 (±0.62) out of 5. More specifically, this outcome was obtained by the mean of all answers concerning the format, utility, interest, and trust given by the study participants, with respect to the online course.

In relation to the evaluation of the separate modules that compose the online course, all six modules were individually graded with a median score of 4.0 or above regarding their adequacy, correction, and completeness, which reflects some homogeneity of the answers among physicians (Table 1). When analyzing the overall evaluation of the modules, the data obtained revealed that all three parameters (adequacy, correction, and completeness) presented a median score of 5 (Table 1).

Overall, the clinical cases received a median score of 5 in all characteristics, with the exception of the parameter’s adequacy and completeness in clinical cases 1 and 2, where a median score of 4.5 and 4.0 were respectively achieved (Table 1).

### 3.3. Evaluation of the eHealthResp Mobile Application

Afterwards, the global evaluation of the clinical decision support system parameters was also carried out by the physicians, with data being shown in Table 2.

The data obtained from the questionnaire unveiled an overall mean score of 4.42 (±0.78) out of 5 for the mobile application assessment. More specifically, this outcome was obtained by calculating the mean of all answers concerning the following seven parameters: adequacy, correction, completeness, format, utility, interest, and trust, given by the study participants, with respect to the mobile application.

Adequacy, correction, format, and trust were graded with the highest median score (5 out of 5), while completeness, utility, and interest were graded with the lowest median score (4 out of 5) (Table 2). Most of the scores obtained from the mobile application assessment revealed a similar distribution of the analyzed characteristics among the physicians under study.

### 3.4. Comparison between the eHealthResp Online Course and Mobile Application

**Figure 1:**
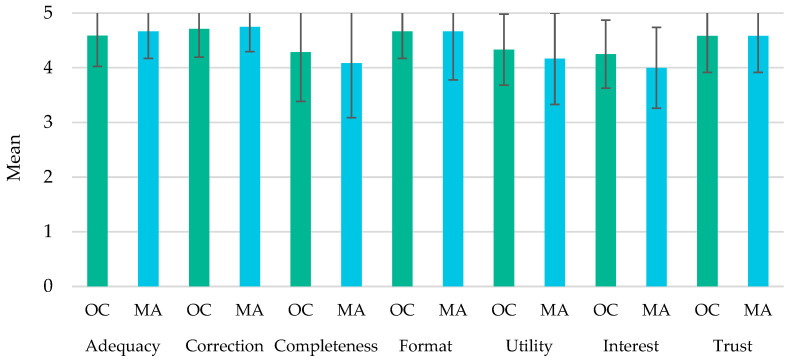
Comparison of the online course (OC) and mobile application (MA) in relation to the seven analyzed parameters (adequacy, correction, completeness, format, utility, interest, and trust). Data are presented as mean values ± standard deviation.

Afterwards, a global comparison between the main characteristics evaluated by the physicians was performed, aiming to assess the major differences between the two digital and powerful platforms. Figure 1 illustrates the overall average scores (±SD) of the assessed parameters.

No major significant differences were found between the online course and mobile application in relation to their adequacy, correction, completeness, format, utility, interest, and trust. While the parameter correction was the one displaying the highest mean score (4.71 for the OC and 4.75 for the MA, out of 5), followed by format and adequacy, the parameter interest was the one displaying the lowest mean score (4.25 for the OC and 4.00 for the MA). Nevertheless, the average overall score reached very high levels in all characteristics under study.

### 3.5. Qualitative Evaluation of Physicians’ Feedback

To complement the quantitative data, all participants also had to undertake a few open-answer questions for a qualitative analysis. These questions ranged from what they liked most or least about the course and mobile application, to what could be improved, and if they indeed benefited from the use of the online course and mobile app. The answers were mostly positive, highlighting the relevance, clarity, easy use, and consolidation of the information presented. However, some physicians considered that additional information could be added, aiming to improve the digital platforms’ contents.

In relation to the question “Considering the main goal of the study, do you think that, in general, this multifaceted educational intervention could be effective in improving the quality of antibiotic prescribing?”, physicians unanimously agreed that this intervention stimulates critical thinking and aids the clinical decision process, avoiding the uncertainty of a diagnosis. Nevertheless, additional training should also be given to health professionals to help increase the impact of these interventions in the combat against antibiotic resistance.

## 4. Discussion

The current pilot study arises as an essential approach to the implementation of a multifaceted educational intervention composed of the eHealthResp online course and mobile application directed to primary care physicians. The main goal is to address one of the top 10 most serious public health threats worldwide [3], ABR, by raising awareness of health professionals about the development and spreading of bacterial resistance due to inappropriate antibiotic prescribing, helping them to improve antibiotic prescription for respiratory tract infections and promoting health literacy.

In general, the evaluation provided by the 12 physicians for both the online course and mobile application was highly positive, with median scores above 4 for all analyzed parameters, thus disclosing the consistency, relevance, and user-friendliness of the eHealthResp platforms. In particular, the most valuable characteristics enhanced by the study participants were related to the format, trust, adequacy, and correction of these e-health tools.

Digital health tools have been widely used by health professionals in medical practice over the last years, as they have the potential to significantly improve the efficiency, accessibility, and quality of care in health systems [26,27,28,29], including antibiotic prescription among healthcare practitioners in primary care [29,30]. Our pilot study has also shown that all physicians unanimously agreed that the tools here presented, namely the online course and mobile application, which constitute a major part of the educational intervention, may also be very efficient in improving the quality of antibiotic prescription and avoiding the uncertainty of a clinical diagnosis, as long as all stakeholders involved are aware of their capabilities and limitations. This will allow a proper scale up of the educational intervention following this study.

Previously published pilot studies have also assessed the potential and utility of e-health tools, such as clinical decision support systems, electronic health records, and online learning by health care professionals, with a sample size similar to ours [31,32,33]. In 2007, Linder et al. [31] designed the acute respiratory infection smart form, a documentation-based clinical decision support system to reduce inadequate antibiotic prescription and improve workflow, which was tested by 10 clinicians. The data revealed the potential of this tool to decrease antibiotic prescription rates and save time, thus being recommended to other professionals [31,34].

This study presents several strengths that should be pointed out, such as the interactivity of both the course and the mobile app, allowing its use with a laptop, mobile phone, or tablet; the improvements made by several experts through the eHealthResp platform’s development process; the physicians’ medical experience; and the homogeneity and positive evaluation provided, thus reflecting the importance of these e-health tools in the optimization of antibiotic prescription for respiratory tract infections.

The main limitation of this study is related to the size of the sample. However, although a higher number of physicians could have potentially given more insightful feedback to improve the eHealthResp platform, 12 participants have shown to be a reasonable number for a pilot study, by taking into consideration previously published studies [35,36,37,38,39]. Moreover, as this study was conducted during the COVID-19 pandemic, one must consider that most health professionals were part of the main workforce, continuously combatting the pandemic effects, thus being unavailable to participate in the study.

Nonetheless, the highly positive feedback obtained in this study also emerges as the result of previous validation studies performed on the eHealthResp platform [22,24,25].

## 5. Conclusions

In sum, the outcomes obtained in this pilot study strongly highlight the quality, user-friendliness, relevance, clarity, and utility of the online course and mobile application, together with a knowledge consolidation by the pilot physicians. The data here presented are very important to assess the feasibility of performing future antibiotic stewardship interventions, namely digital-based interventions, with a larger group of physicians, tailored to improve the quality of prescription practices in respiratory infections. Furthermore, this study can serve as a reference to other pilot studies, as more initiatives are needed to increase the impact on this public health problem, considering that a good dissemination of information by the various health professionals is required.

## Figures and Tables

**Table 1:** Physicians’ evaluation of the overall characteristics’ contents and clinical cases of the eHealthResp online course.

**Online Course**	**Parameters**	**Median (PCT25, PCT75)**
	Format	5.00 (4.00, 5.00)
	Utility	4.00 (4.00, 5.00)
	Interest	4.00 (4.00, 5.00)
	Trust	5.00 (4.00, 5.00)
**Modules**	**Parameters**	**Median (PCT25, PCT75)**
	Adequacy	4.50 (4.00, 5.00)
Module 0	Correction	5.00 (4.00, 5.00)
	Completeness	4.00 (3.75, 5.00)
	Adequacy	5.00 (4.00, 5.00)
Module 1	Correction	5.00 (5.00, 5.00)
	Completeness	4.00 (4.00, 5.00)
	Adequacy	5.00 (4.00, 5.00)
Module 2	Correction	5.00 (4.75, 5.00)
	Completeness	5.00 (4.00, 5.00)
	Adequacy	5.00 (4.75, 5.00)
Module 3	Correction	5.00 (4.75, 5.00)
	Completeness	5.00 (4.00, 5.00)
	Adequacy	5.00 (4.00, 5.00)
Module 4	Correction	5.00 (5.00, 5.00)
	Completeness	5.00 (4.00, 5.00)
	Adequacy	5.00 (4.75, 5.00)
Module 5	Correction	5.00 (4.75, 5.00)
	Completeness	4.50 (4.00, 5.00)
	Adequacy	5.00 (4.00, 5.00)
Module 6	Correction	4.50 (4.00, 5.00)
	Completeness	4.00 (3.00, 5.00)
**Clinical Cases**	**Parameters**	**Median (PCT25, PCT75)**
	Adequacy	4.50 (4.00, 5.00)
Clinical Case 1	Correction	5.00 (4.75, 5.00)
	Completeness	4.00 (4.00, 5.00)
	Adequacy	4.50 (4.00, 5.00)
Clinical Case 2	Correction	5.00 (4.00, 5.00)
	Completeness	4.00 (4.00, 5.00)
	Adequacy	5.00 (5.00, 5.00)
Clinical Case 3	Correction	5.00 (5.00, 5.00)
	Completeness	5.00 (4.00, 5.00)
	Adequacy	5.00 (4.00, 5.00)
Clinical Case 4	Correction	5.00 (4.75, 5.00)
	Completeness	5.00 (4.00, 5.00)

**Table 2:** Physicians’ evaluation of the overall characteristics of the eHealthResp mobile application.

Mobile App	Parameters	Median (PCT25, PCT75)
	Adequacy	5.00 (4.00, 5.00)
	Correction	5.00 (4.75, 5.00)
	Completeness	4.00 (3.75, 5.00)
	Format	5.00 (5.00, 5.00)
	Utility	4.00 (4.00, 5.00)
	Interest	4.00 (3.75, 4.25)
	Trust	5.00 (4.00, 5.00)

## Data Availability

Not applicable.

## Supplementary Materials

The following supporting information can be downloaded at: https://www.mdpi.com/article/10.3390/life12081160/s1. Supplementary Material S1: eHealthResp Online Course Platform; Supplementary Material S2: eHealthResp Mobile Application Platform.
